# 以获得性血管性血友病综合征发病的弥漫大B细胞淋巴瘤1例报告并文献复习

**DOI:** 10.3760/cma.j.cn121090-20250312-00130

**Published:** 2025-11

**Authors:** 烽迪 王, 冰洁 丁, 梦娟 李, 雪雯 宋, 俊霞 胡, 柳 刘, 虎 周

**Affiliations:** 1 郑州大学附属肿瘤医院（河南省肿瘤医院），郑州 450008 The Affiliated Cancer Hospital of Zhengzhou University & Henan Cancer Hospital, Zhengzhou 450008, China; 2 郑州大学第一附属医院，郑州 450002 The First Affiliated Hospital of Zhengzhou University, Zhengzhou 450052, China

## Abstract

本文报告1例以获得性血管性血友病综合征（AvWS）为首发症状的弥漫大B细胞淋巴瘤（DLBCL）患者的诊治过程，并结合文献进行复习。患者为22岁男性，因反复黏膜出血就诊，初期诊断为遗传性血管性血友病，后因胸壁包块确诊为DLBCL（非生发中心型，Ann Arbor分期Ⅳ期）。通过化疗联合自体造血干细胞移植及泽布替尼维持治疗，患者实现了持续完全缓解，且凝血功能恢复正常。本病例为淋巴瘤相关AvWS的诊疗提供了重要参考，且凸显早期识别基础疾病的重要性。

## Abstract (en)

This article reports the diagnosis and treatment of a patient with diffuse large B-cell lymphoma（DLBCL）initially manifested as acquired von Willebrand syndrome（AvWS）, along with a literature review. The patient, a 22-year-old male, was diagnosed with hereditary von Willebrand disease at the initial stage due to repeated mucosal bleeding, and was later diagnosed with DLBCL（non germinal center type, Ann Arbor stage Ⅳ）due to chest wall mass. Through chemotherapy combined with autologous hematopoietic stem cell transplantation and zebutinib maintenance therapy, the patient achieved sustained complete remission, and the coagulation function returned to normal. This case provides an important reference for the diagnosis and treatment of lymphoma associated AvWS, and highlights the importance of early recognition of basic diseases.

获得性血管性血友病综合征（acquired von Willebrand syndrome, AvWS）是一种罕见的获得性出血性疾病，其临床和实验室特征与遗传性血管性血友病（von Willebrand disease, vWD）相似，但无个人或家族出血史，多继发于其他基础疾病[1]–[2]。流行病学数据显示，AvWS与淋巴增殖性疾病、骨髓增殖性疾病、心血管疾病等相关，且国内少有报道[3]–[5]。近期本中心成功诊治1例以AvWS为首发症状的弥漫大B细胞淋巴瘤（DLBCL）患者，现报告本病例的诊治过程并复习相关文献。

## 病例资料

患者，男性，22岁，于2023年5月因“间断黏膜出血1年余”首次就诊于郑州大学附属肿瘤医院。患者自2022年4月无明显诱因出现间断牙龈及鼻腔出血，2022年8月就诊于外院查血管性血友病因子（vWF）抗原（vWF∶Ag）<1％，FⅧ活性11.1％，FⅧ抑制物0.06 BU/ml，血常规未见明显异常。2023年5月血常规示：WBC 4.98×10^9^/L，PLT 70×10^9^/L，HGB 127 g/L，活化部分凝血活酶时间（APTT）51.5 s，凝血酶原时间（PT）11.4 s，vWF∶Ag 11％，LDH 430 U/L。结合患者病史及检查结果初步诊断为遗传性vWD，按需输注FⅧ治疗，输注FⅧ后患者出血症状好转，但治疗后未复查vWF∶Ag。2023年6月患者因发现胸壁包块逐渐增大再次就诊于我院，胸部增强CT提示胸骨骨质破坏伴周围稍高密度影，符合血友病改变。患者有慢性乙型病毒性肝炎病史，无家族史。查体示胸前壁可见7 cm×5 cm包块，边界清晰，无红肿，有压痛。入院后进一步完善相关检查，实验室检查提示：WBC 3.96×10^9^/L，PLT 41×10^9^/L，HGB 84 g/L，APTT 50.7 s，FⅧ活性5％。完善全身PCT-CT提示：锁骨、纵隔、腹腔、肝门等多发软组织影，代谢增高，恶性病变（淋巴瘤）可能性大；胸骨局部溶骨性骨质破坏并软组织影，代谢增高，考虑恶性；余全身多发骨骼代谢增高，局部呈溶骨性骨质破坏，恶性病变侵及可能性大。根据PET-CT结果进一步完善骨髓穿刺术，骨髓细胞学提示：骨髓有核细胞增生明显活跃，淋巴细胞比例增高（54.4％），其中幼淋样细胞占25.6％，余各系未见明显异常。外周血涂片可见3％异常幼淋样细胞。流式细胞术检测免疫表型提示：异常单克隆性成熟B淋巴细胞群，占有核细胞26.2％，免疫表型符合CD5^-^ CD10^-^ 成熟B细胞淋巴瘤表型。染色体核型分析：46，XY，+2 add（21）（q22）[5]/46，XY[4]。二代测序检测基因突变阴性。骨髓活检免疫组化考虑DLBCL累及。因凝血功能异常为淋巴结切除术禁忌证，遂未进一步行完整淋巴结病灶切除病理活检。结合患者现有检查结果及病史，诊断为DLBCL（非生发中心型，Ann Arbor分期Ⅳ期）。按照“一元论”的基本原则，我们怀疑患者近期凝血功能异常可能由DLBCL继发AvWS导致。明确诊断后立即行R-CHOP方案化疗，第1个周期化疗期间患者间断有鼻衄，按需输注FⅧ纠正患者凝血功能异常，患者凝血功能好转，未予其他特殊治疗。第2个周期化疗开始前患者APTT、vWF活性及FⅧ活性均已恢复正常，后续治疗期间持续监测凝血功能、vWF活性及FⅧ活性均处于正常范围，未再次出血，亦未再次输注FⅧ，进一步证实了AvWS的诊断。患者共接受3个周期R-CHOP（利妥昔单抗+环磷酰胺+表阿霉素+长春新碱+地塞米松）方案和1个周期R+DA-EPOCH（利妥昔单抗+依托泊苷+长春地辛+阿霉素+环磷酰胺+地塞米松）方案，PET-CT及骨髓细胞学评估疾病为完全缓解（CR）。随后患者行自体造血干细胞动员及采集，采集后再次接受3个周期Pola-R-CHP（维泊妥珠单抗+利妥昔单抗+环磷酰胺+表阿霉素+地塞米松）方案，再次评估疾病仍为CR。2024年1月患者接受自体造血干细胞移植（auto-HSCT），移植预处理方案为BEAM（司莫司汀+阿糖胞苷+依托泊苷+美法仑），回输过程平稳，患者血细胞计数恢复后出院。auto-HSCT后患者持续口服泽布替尼160 mg每日2次维持治疗。自2024年2月随访至2025年3月，患者仍处于CR状态，凝血功能、vWF活性及FⅧ活性均正常。

## 讨论及文献复习

AvWS是一种罕见的获得性出血性疾病，文献提示其发病率约占所有出血性疾病的3％[6]。自50余年前首次报道以来[7]，AvWS逐渐被临床医师关注。早年的流行病学数据显示，AvWS常与淋巴增殖性疾病（如慢性淋巴细胞白血病、毛细胞白血病和非霍奇金淋巴瘤等，占48％）、骨髓增殖性肿瘤［MPN，如原发性血小板增多症（ET）和真性红细胞增多症（PV），占15％］、单克隆丙种球蛋白病（如华氏巨球蛋白血症、意义未明的单克隆丙种球蛋白血症和多发性骨髓瘤）、实体恶性肿瘤（占5％）、心血管疾病（如主动脉瓣狭窄和左心室安装辅助装置者，占21％）和自身免疫性疾病（占2％）等相关[1]。而近年来的研究表明，AvWS发病率可能被低估，尤其是在心血管疾病和血液系统恶性肿瘤患者中[8]–[11]。Icheva等[8]的回顾性研究显示，在12例接受手术的复杂先天性心脏病婴幼儿中，8例（66％）患儿在术中接受体外循环后被诊断为AvWS。Rottenstreich等[11]则发现，在173例MPN患者中，55％的ET和49％的PV患者在病程中曾发生AvWS。与遗传性vWD相比，AvWS患者多于成年发病，无家族出血史，常伴随上述基础疾病。AvWS患者的出血症状以皮肤黏膜为主（鼻出血、牙龈出血），偶见深部组织出血或术后出血。

研究表明，AvWS的发病机制复杂多样，主要涉及vWF的加速清除、功能异常或生成减少[12]。其核心机制包括以下几个方面：①自身抗体介导的清除：在淋巴增殖性疾病和自身免疫性疾病中，患者体内可产生针对vWF的自身抗体（IgG或IgM）。这些抗体通过与vWF结合形成免疫复合物加速vWF的清除，或直接中和其功能域（如GPⅠb结合区），导致vWF活性丧失[13]。②肿瘤细胞吸附：部分实体瘤或血液系统恶性肿瘤细胞表面异常表达vWF受体（如GPⅠb或GPⅡb/Ⅲa），选择性吸附高分子量vWF多聚体，导致血浆中功能性vWF减少[14]。③剪切力诱导的降解：在主动脉瓣狭窄或左心室辅助装置患者中，异常血流剪切力导致vWF多聚体结构展开，易被ADAMTS13蛋白酶降解[15]。④血小板介导的清除：MPN（如ET）患者血小板数量显著升高，通过吸附或蛋白酶作用加速vWF降解[16]。⑤vWF生成减少：在甲状腺功能减退患者中，vWF的生成显著减少[17]。值得注意的是，部分病例可能同时存在多种机制，如单克隆丙种球蛋白病患者既可产生抗vWF抗体，又因异常浆细胞表面受体吸附vWF。

诊断AvWS的实验室检查包括：①瑞斯托霉素辅因子活性（vWF∶RCo）和胶原结合活性（vWF∶CB）显著低于vWF∶Ag，通常vWF∶RCo/vWF∶Ag<0.7。②高分子量vWF多聚体缺失。③FⅧ活性降低，APTT轻度延长。另外诊断需排除遗传性vWD，尤其关注无家族史、迟发出血、合并前述基础疾病的患者[18]。

AvWS的治疗根本上是针对原发病的治疗。除此之外，患者有急性出血时，可选择去氨加压素（DDAVP），但疗效短暂，成功率约30％，且需密切监测vWF水平。DDAVP无效者可输注vWF/FⅧ浓缩物。难治性出血可输注重组活化因子Ⅶ（rFⅦa）激活旁路途径进行止血。也可将抗纤溶药物与DDAVP或vWF/FⅧ浓缩物联用，用于预防出血或治疗黏膜出血。大剂量免疫球蛋白静脉冲击治疗（HD-IVIg）可用于中和vWF抑制物，必要时可尝试血浆置换以快速清除自身抗体或单克隆免疫球蛋白[9]。

本例患者为22岁男性，无家族史，以自发口腔、鼻腔黏膜出血起病，多次就诊于外院，均诊断为遗传性vWD，按需给予FⅧ治疗可改善出血症状，近1年无其他特殊不适。2023年5月初次就诊于我院时，患者已有血小板轻度减少、LDH升高等不符合遗传性vWD的临床表现，且患者已出现胸壁包块，但由于包块较小，患者未主动提及，导致遗漏这一重要体征。根据患者vWF∶Ag和FⅧ活性显著降低，诊断为遗传性vWD，按需给予FⅧ治疗。患者在外院和本院均仅检测vWF∶Ag，未检测vWF∶RCo和vWF∶CB，也是本病例初诊时误诊的重要原因之一。2023年6月患者自觉胸壁包块逐渐增大再次就诊，此时患者已出现中度贫血和中度血小板减少，进一步检查确诊DLBCL。本例患者发病过程体现了AvWS与恶性血液病关联的典型特征，并凸显了在无家族遗传史患者中识别继发性凝血障碍的重要性，需要积极筛查潜在基础疾病。因患者诊断时处于Ann Arbor分期Ⅳ期，确诊后立即给予标准R-CHOP方案治疗，患者APTT、vWF活性及FⅧ活性在1个月内迅速恢复正常，后复查凝血功能、FⅧ活性、vWF活性均在正常范围内，更加支持AvWS而非遗传性vWD的诊断。患者CR后接受auto-HSCT，移植后持续口服泽布替尼维持治疗，最终实现持续CR。因国内外报道DLBCL继发AvWS的病例极少，R-CHOP方案作为DLBCL一线治疗方案对AvWS是否有直接治疗作用尚不清楚。但AvWS的发病机制中存在自身免疫机制，R-CHOP方案中的CD20单抗、环磷酰胺、长春新碱和泼尼松均有免疫抑制作用，这些药物同时也是原发免疫性血小板减少症、自身免疫性溶血性贫血、获得性血友病等血液科自身免疫性疾病的常用药物。因此我们推断此方案不仅诱导淋巴瘤缓解，消除了AvWS的诱发因素，可能也对AvWS有直接治疗作用。但根治原发病仍是治疗AvWS的核心与根本，若未及时识别原发病，单纯依赖凝血因子替代治疗可能延误病因治疗，导致病情进展。

AvWS作为非霍奇金淋巴瘤的罕见并发症，常以出血为首发表现，易被误诊为遗传性vWD。对于无家族史、新发黏膜出血的年轻患者，需高度警惕继发性AvWS，尤其需排查淋巴瘤、MPN等血液系统恶性疾病。本病例中诱发AvWS的是恶性淋巴瘤，随着学科亚专科的逐渐精细化，需要淋巴瘤亚专科和出凝血亚专科之间积极合作和共同诊治。对于其他非血液病诱发的AvWS，多学科合作更加重要。在AvWS的管理中，病因探索与精准治疗是改善预后的基石。本例患者通过全面检查明确诊断为DLBCL，并通过强化化疗联合auto-HSCT实现疾病CR，为类似病例提供了参考。未来需进一步研究淋巴瘤相关AvWS的分子机制，以优化个体化治疗策略。
